# Mother-to-Child HTLV-1 Transmission: Unmet Research Needs

**DOI:** 10.3389/fmicb.2019.00999

**Published:** 2019-05-08

**Authors:** Carolina Rosadas, Graham P. Taylor

**Affiliations:** Retrovirology and GU Medicine, Department of Medicine, Imperial College London, London, United Kingdom

**Keywords:** HTLV-1, mother-to-child transmission, pregnancy, outcomes, risk, disease

## Abstract

Mother-to-child transmission (MTCT) of Human T-cell lymphotropic virus type 1 (HTLV-1) causes lifelong infection. At least 5–10 million individuals worldwide are currently living with HTLV-1. Studies of regional variation are required to better understand the contribution of MTCT to the global burden of infection. Although most infected individuals remain asymptomatic ∼10% develop high morbidity, high mortality disease. Infection early in life is associated with a higher risk of disease development. Adult T-cell leukemia (ATL), which is caused by HTLV-1 and has a median survival of 8 months is linked to MTCT, indeed evidence of ATL following infection as an adult is sparse. Infective dermatitis also only occurs following neonatal infection. Whilst HTLV-1-associated myelopathy (HAM) follows sexual and iatrogenic infection approximately 30% of patients presenting with HAM/TSP acquired the infection through their mothers. HAM/TSP is a disabling neurodegenerative disease that greatly impact patient’s quality of life. To date there is no cure for HTLV-1 infection other than bone marrow transplantation for ATL nor any measure to prevent HTLV-1 associated diseases in an infected individual. In this context, prevention of MTCT is expected to contribute disproportionately to reducing both the incidence of HTLV-1 and the burden of HTLV-1 associated diseases. In order to successfully avoid HTLV-1 MTCT, it is important to understand all the variables involved in this route of infection. Questions remain regarding frequency and risk factors for *in utero* peri-partum transmission whilst little is known about the efficacy of pre-labor cesarean section to reduce these infections. Understanding the contribution of peripartum infection to the burden of disease will be important to gauge the risk-benefit of interventions in this area. Few studies have examined the impact of HTLV-1 infection on fertility or pregnancy outcomes nor the susceptibility of the mother to infection during pregnancy and lactation. Whilst breast-feeding is strongly associated with transmission and avoidance of breast-feeding a proven intervention little is known about the mechanism of transmission from the breast milk to the infant and there have been no clinical trials of antiretroviral therapy (ARV) to prevent this route of transmission.

## Introduction

Human T-cell lymphotropic virus type 1 (HTLV-1) is a human retrovirus that is mostly transmitted through sexual intercourse and from mother-to-child. Early after the first identification of HTLV-1 the possibility of mother-to-child transmission (MTCT) was supported by several pieces of evidence including: (1) high incidence of HTLV-1 infection in children born to HTLV infected mothers; (2) high prevalence of infection in mothers of HTLV-1 seropositive children; (3) presence of approximately 10^3^ cells infected with HTLV/mL in milk from carrier mothers; (4) experimental infection in animals using fresh milk cells from HTLV-1 infected women (Hino, 2011); (5) low transmission risk among bottle-fed children when comparing to breast fed; and more recently reduced incidence of infection after implementation of antenatal screening and avoidance of breastfeeding observed in Japan. Thus, this route of infection is well stablished and is responsible for the maintenance of HTLV-1 in several generations of the same family (The T and B-cell malignancy study group, 1988; Plancoulaine et al., 1998; Catalan-Soares et al., 2005; da Costa et al., 2013; Mendes et al., 2016). Moreover, transmission is known to be associated with the risk of adult T-cell leukemia (ATL), a highly aggressive and usually fatal malignancy caused by HTLV-1. Despite almost 40 years passing since the discovery of HTLV-1 many gaps in our understanding of MTCT remain. This review aims to summarize what is known, the current limitations of this knowledge and to identify the most important areas for further research.

## HTLV-1 Infection in Pregnant Women

What We Need to Know About HTLV-1 Infection in Pregnant Women

• What is the prevalence of HTLV-1 infection in pregnant women in different countries/regions?
• What is the susceptibility to HTLV-1 infection during pregnancy and lactation?
• What is the impact of pregnancy on HTLV-1 infection – is viral control affected?
• What is the impact of pregnancy in HTLV-1 associated diseases and *vice-versa*?
• Does HTLV-1 infection impact fertility, miscarriage rates or 3rd trimester pregnancy outcomes including birth weight and gestational age at delivery?
• What is the impact of HTLV-1 infection and/or its diagnosis on psychological health of pregnant women and parturient?

### Prevalence of HTLV-1 Infection in Pregnant Women

HTLV-1 prevalence is considered more representative of the general population than prevalence studies among blood donors due to the impact of pre-screening on donor self-selection. However, many cross-sectional studies also show a significant increase in prevalence with age, particularly in women and most notably after age 40 (Plancoulaine et al., 1998). This is considered to result from a birth cohort effect as well as horizontal transmission (Satake et al., 2015). Therefore younger women of reproductive age are not fully representative and prevalence is likely to be underestimated in the overall population (Proietti et al., 2005). However, HTLV-1 prevalence among pregnant women is useful to indicate the regions where implementation of measures to prevent MTCT is urgent.

**FIGURE 1:**
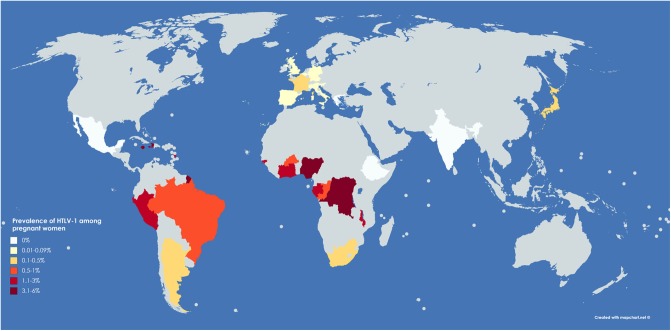
Representative map of world prevalence of HTLV-1 in pregnant women. Original figure created with mapchart.net. Areas in pale gray are those with unknown HTLV-1 prevalence in pregnant women.

The published prevalence of HTLV-1 infection among pregnant women is represented in the map (Figure 1) and presented in Supplementary Table. Although some studies are not comparable, due to differences in methodologies, the overall picture shows wide variation not only between but within countries. Importantly, many regions lack any data about HTLV-1 prevalence in this population whilst, even where the prevalence has been reported, many studies are out of date and were conducted with less specific assays. To better understand the public health importance of HTLV-1 infection new, adequately powered studies examining HTLV-1 seroprevalence in most regions of the world are a priority.

### Susceptibility to HTLV-1 Infection During Pregnancy and Lactation

**FIGURE 2:**
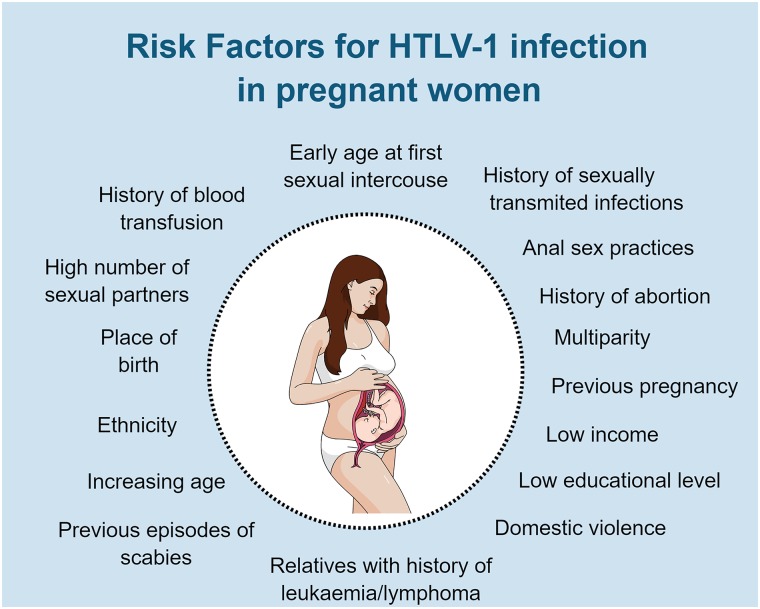
Risk factors for HTLV-1 infection in pregnant women or women at reproductive age. Original figure created with mindthegraph.

The various risk factors for HTLV-1 infection that have been reported in pregnant women or in women of reproductive age are listed in Figure 2 (Sanchez-Palacios et al., 2003; Alarcón et al., 2006; Moxoto et al., 2007; Dal Fabbro et al., 2008; Etenna et al., 2008; Lima and Viana, 2009; Ydy et al., 2009; Ribeiro et al., 2010). Most studies did not perform multivariate analysis to confirm the risk factors. Increasing age and age at first sexual intercourse were strongly associated with HTLV-1 infection, while history of abortion and history of transfusion presented borderline statistical significance in a multivariate analysis (Alarcón et al., 2006). These risk factors were observed in pregnant women assuming that infection occurred previously. An increase in susceptibility to HIV infection during pregnancy and lactation is reported (Thomson et al., 2018), but there are no similar studies in HTLV-1. Therefore, we do not know if pregnancy and lactation impact the susceptibility to HTLV-1 infection.

### The Impact of Pregnancy on HTLV-1 Infection Control

Few studies have examined the impact of pregnancy on HTLV-1 infection. HTLV-1 proviral load (PVL), which is until now, the main predictor of disease outcome, seems to be stable during pregnancy, but increase after delivery in a recent Japanese study of 36 women (Fuchi et al., 2018). However, HTLV-1 PVL before conception was not known. It is assumed that these women had chronic infection. There are limited data on the kinetics of HTLV-1 infection but in one detailed study in a transplant setting, PVL increased rapidly during the first few weeks and had reached steady state by 38–45 days after exposure (Cook et al., 2016). Since pregnancy represents a state of relative immunosupression it would be important to know whether the rate of viral replication and the steady state PVL differ when infection is first acquired during pregnancy, as this would likely impact on HTLV-1 MTCT. Whether a similar pattern of change in PVL with pregnancy is observed in different regions, in populations with different genetic background and in different environments, where for example, there is a high prevalence of infectious agents or different socio-economic conditions needs to be determined. Similarly, if PVL rises after pregnancy is this transient or sustained and do serial pregnancies matter?

### The Impact of Pregnancy on HTLV-1 Associated Diseases and *vice-versa*

There are few data on the impact of pregnancy on the progression of HTLV-1 associated diseases or *vice-versa*. ATL in pregnancy has been reported. The majority were acute ATL and most patients died soon after delivery (by pre labor cesarean section [PLCS]), despite treatment (Utsumi et al., 1996; Safdar et al., 2002; Amor et al., 2013). Interestingly, there is one report of chronic ATL in a pregnant woman, previous diagnosed as asymptomatic carrier (AC). In this case, chronic ATL with unfavorable prognosis (aggressive ATL) was diagnosed at 12 weeks of pregnancy. The pregnancy was terminated at 15 weeks following which the unfavorable factors (high lactate dehydrogenase (LDH) and low serum albumin) improved, with re-classification to favorable (indolent ATL) and a watchful waiting strategy was adopted (Fuchi et al., 2016). Interestingly, Fuchi et al. (2018) also identified higher frequency of CD4^+^CD25^+^CD127^low/-^ cells and increased sIL-2R in HTLV-1 infected women compared with un-infected women 1 month after delivery in their recent study. The optimal management of HTLV-1 infection and ATL in this setting is not known. Three cases from the United Kingdom were presented in 2017. MTCT despite PLCS occurred in the first case whilst zidovudine and raltegravir were added to maternal treatment along with neonatal zidovudine and PLCS for HTLV-1 MTCT in the subsequent cases – with no evidence of transmission at last follow-up (Cook et al., 2017).

Although no studies regarding HAM/TSP in pregnant women have been published there is clear potential for pregnancy to impact HAM, directly or indirectly. HAM/TSP is an inflammatory disease and the severity of many diseases, particularly autoimmune, is reduced during pregnancy due to the change in Th1/Th2 balance (Mor and Cardenas, 2010). Conversely, bladder and bowels symptoms such as frequency and constipation are common in both HAM and pregnancy whilst gait can be expected to worsen due to the physiological changes in pregnancy. Neurogenic bladder which is commonly observed in HAM/TSP (Araujo, 2015), could impact urinary infection rate (Nomata et al., 1992), which, in turn, can induce preterm birth (Dautt-Leyva et al., 2018).

### Impact of HTLV-1 Infection in Miscarriage Rates and 3rd Trimester Pregnancy Outcomes

A number of studies have explored the impact of HTLV-1 infection on pregnancy. In a study from Mato Grosso do Sul (Brazil) (Dal Fabbro et al., 2008) 6.7% (11/164) of HTLV-1 associated pregnancies resulted in first trimester miscarriage. Moreover, HTLV-1 infected pregnant women reported, more frequently [26.8% (41/153)], history of previous miscarriage than non-infected women (16%). Similar results were observed in Peru, where women with a history of abortion were more likely to be infected with HTLV-1 [prevalence of HTLV-1 infection 3.2% vs. 1.1%, Crude OR (95% CI): 3 (1.8–5.0)] (Alarcón et al., 2006). In the same study, the prevalence of HTLV-1 infection was higher among those women who presented at the hospital for abortion/miscarriage (3.5%) than for prenatal (1.4%) or post-natal care (1.7%) (Alarcón et al., 2006). As induced abortion is illegal in Peru, it is not possible to elicit any conclusion about those cases of abortions/miscarriage. Moreover, multivariate analysis was not performed to check for confounding factors such as the presence of co-infections. It is known that approximately half of all preterm deliveries are associated with histologic evidence of placental inflammation (acute chorioamnionitis (ACA) or chronic chorioamnionitis), regardless of fetus infection (Mor and Cardenas, 2010). Underweight babies, pre-term and higher numbers of hospitalization was observed among HTLV-1 infected children (*n* = 58) when comparing to non-infected children born from seropositive mothers (*n* = 42) and may raise the question if underweight babies or preterm births are risk factors for increased HTLV-1 susceptibility (Kendall et al., 2009). In a case-control study in Gabon of 45 HTLV-1 infected mothers each matched to two controls higher rates of preterm delivery (11% vs. 2.7%), complicated pregnancy (36.5% vs. 22%) and cesarean sections (8% vs. 3%) were observed in HTLV-1 infected women compared to the control group, but none of these differences were statistically significant (Ville et al., 1991). Moreover, other studies (of up to 45 HTLV-1 infected women) did not observe any impact of HTLV-1 infection on miscarriage or preterm delivery rates (Saito et al., 1990; Bittencourt et al., 2001; Montano et al., 2004). Clearly the small sample size of these studies precludes definitive conclusions to be drawn and more research regarding this topic is needed. It is also important to understand which mechanisms might be involved in the impact of this virus in pregnancy. HTLV-1 infection is known to be associated with an immune imbalance, characterized mainly by the production of pro-inflammatory cytokines, more evident in persons with HAM/TSP (Futsch et al., 2018; Rosa et al., 2018) As pro-inflammatory mediators have been already associated with unfavorable pregnancy outcomes in HIV infection (Pfeifer and Bunders, 2016), it is plausible to assume that HTLV-1 infection may also be associated with negative pregnancy outcomes.

### HTLV-1 Infection and Fertility

There are limited data on the impact of HTLV-1 on fertility and on assisted reproduction. While HTLV-1 infection can be associated with erectile dysfunction in up to 55.2% men (Caskey et al., 2007; Oliveira et al., 2010; de Oliveira et al., 2017) and with sexual dysfunction in women (Lopes Martins et al., 2018), no differences in the prevalence of HTLV-1 infection according to the fertility status was observed in women from Gabon (Schrijvers et al., 1991). One study about assisted reproduction from Iran, evaluated 32 cycles from HTLV-1 positive women and when comparing to an age matched control group no differences were found among fertilization, implantations, pregnancy rate, number of transferred and cryopreserved embryos, multiple pregnancies nor in abortion rates (Torshizi et al., 2014). However, a question that is raised is: should assisted reproduction be indicated for HTLV discordant couples? Moreover, testing couples that seek assisted reproduction is also important (Epelboin, 2011). Indeed, in the United Kingdom, in accordance with European Regulations, the donors of all tissue used in therapy, including assisted reproduction are tested for HTLV-1 infection (ECDC, 2012). In Brazil, HTLV screening in germinative cells donors and patients seeking for assisted reproduction is also implemented (ANVISA, 2011).

### Impact of HTLV-1 on Psychological Health of Pregnant Women and Parturient

Another point that needs more attention is about the impact of HTLV-1 infection in the psychological health of pregnant women and parturient (Carneiro-Proietti et al., 2014). Pregnancy and birth are unique moments, when women already must face many changes. Little is known about the impact of HTLV-1 infection and/or the diagnosis on the emotional condition of mothers to be (anxiety, depression), parturient nor on the relationship among mothers/babies or with their family members. Clinical experience suggests that various factors impact on this: knowledge and perception of HTLV-1 infection as well as timing of diagnosis. Some have cited anxiety as a reason not to screen for HTLV-1 infection ante-natally referring to a single paper from Japan (UK National Screening Committee, 2017). It should, however, be noted that a national antenatal screening programme for HTLV-1 has been successfully introduced in Japan. There, pregnant women were tested during the third trimester in order to minimize their anxiety and prevent artificial abortions (Hino, 2011).

## HTLV-1 Mother to Child Transmission (MTCT)

What we need to know about HTLV-1 mother-to-child transmission

• Does intra-uterine transmission occur? If so, why it is so rare?
• Which mechanisms are involved in the protection of intra-uterine transmission?
• What factors are important in HTLV-1 MTCT?
• What is the impact, if any, of co-infections in the risk of MTCT?
• Which cells are involved with HTLV-1 transmission via oral route?
• What is the mechanism of HTLV-1 transmission across the digestive tract?
• Is the cellular component in breast milk from HTLV-1 infected women similar to healthy uninfected women over time?
• What is the role of different breast milk constituents in the efficiency of infection?
• There is a need to identify maternal neutralizing antibodies and their role in protection.

### What Is Known About Intra-uterine Transmission?

The overall MTCT rates observed in different areas are presented in Table 1. There is substantial evidence that ante-natal and perinatal infection are less frequent than transmission through breastfeeding, as summarized by Hino (2011). The low level of infection among babies that are formula fed is one of the most compelling evidence (Table 2; Ando et al., 1987, 2003, 2004; Biggar et al., 2006; Bittencourt et al., 2002; Del Mistro et al., 1994; Gotuzzo et al., 2007; Hamedi et al., 2012; Hino et al., 1985; Hisada et al., 2002; Kashiwagi et al., 2004; Kusuhara et al., 1987; Li et al., 2004; Maloney et al., 2003; Monplaisir et al., 1993; Montano et al., 2004; Nyambi et al., 1996; Oki et al., 1992; Paiva et al., 2018; Ribeiro et al., 2012; Takahashi et al., 1991; Tsuji et al., 1990; Ureta-Vidal et al., 1999; Van Tienen et al., 2012; Wiktor et al., 1997; Wiktor et al., 1993).

**Table 1:** Rate of HTLV-1 mother-to-child transmission.

References	Country	HTLV-1 mother-to-child transmission rate (%)
Paiva et al., 2018	Brazil	14.2
Ureta-Vidal et al., 1999	French Guyana	9.7
Nyambi et al., 1996	Gabon	17.5
Del Mistro et al., 1994	Gambia	22
Van Tienen et al., 2012	Guinea-Bissau	25
Hamedi et al., 2012	Iran	16.6
Biggar et al., 2006	Jamaica	17
Barcellos et al., 2005	Jamaica	22
Li et al., 2004	Jamaica	22
Maloney et al., 2003	Jamaica	15.2
Hisada et al., 2002	Jamaica	18
Wiktor et al., 1997	Jamaica	18
Wiktor et al., 1993	Jamaica	23
Kashiwagi et al., 2004	Japan	16
Kashiwagi et al., 2004	Japan	3.9
Takahashi et al., 1991	Japan	8.5
Tsuji et al., 1990	Japan	21
Kusuhara et al., 1987	Japan	15.4
Ando et al., 1987	Japan	46
Hino et al., 1985	Japan	17
Monplaisir et al., 1993	Martinique	27
Gotuzzo et al., 2007	Peru	19
Montano et al., 2004	Peru	18


**Table 2:** Rate of HTLV-1 mother-to-child transmission according to the duration of breastfeeding.

References	Country	HTLV-1 MTCT rate			
		No Breastfeeding	Short-term breastfeeding	Long-term breastfeeding	Freeze-thawed milk
Paiva et al., 2018	Brazil		4.8% (<12 m)	23.8% (>12 m)	
Ureta-Vidal et al., 1999	French Guyana	2.5% (0-3 m)	5.9% (4-6 m)	7% (7-9 m)	
				10.3% (10-12 m)	
				12.9% (>12 m)	
Wiktor et al., 1997	Jamaica		9% (<6 m)	32% (>6 m)	
Wiktor et al., 1993	Jamaica		7% (<6 m)	21% (>6 m)	
Hino, 2011	Japan	2.5%	7.4% (<6 m)	20.3% (>6 m)	
Ando et al., 2004	Japan				0%
Ando et al., 2003	Japan				4.60%
Hino et al., 1996	Japan		6% (<6 m)	13.7% (>6 m)	
				15.7% (>12 m)	
Oki et al., 1992	Japan		4.5% (<7 m)	14% ( > 7 m)	
Oki et al., 1992	Japan	5.6%	3.8% (<7 m)	25% (>7 m)	
Takahashi et al., 1991	Japan	5.7%	4.4% (<6 m)	14.4% (>7 m)	


*m, months.*

HTLV-1 proviral DNA was detected in 2/9 placentas from HTLV-1 positive mothers by nested PCR. HTLV-1 antigen (p19) was also identified in placental villous cells cultured from these women (Fujino et al., 1992). Others, using culture and PCR were unable to detect HTLV-1 infected cells or provirus DNA in cord blood (Hino et al., 1985; Saji et al., 1989, 1990; Tsuji et al., 1990; Nyambi et al., 1996) nor in amniotic fluid (Nyambi et al., 1996). Where identification of HTLV-1 proviral DNA in cord blood was reported (Saito et al., 1990), there was no clear association with new-born infection. In a study from Japan, none of seven children with positive HTLV-1 DNA in cord blood seroconverted while nine that seroconverted did not have HTLV-1 DNA in cord blood (Kawase et al., 1992; Hino et al., 1997). In the other hand, at least two cases of bottle-fed infants, born by PLCS, infected by MTCT were already reported (Saito et al., 1990; Cook et al., 2017). While one baby was born from a mother with ATL (Cook et al., 2017), the other one was Rh positive, born from Rh negative mother with indirect Coombs positive test after 24th weeks of pregnancy (Saito et al., 1990). These observations may indicate transplacental infection. Due to these mixed findings, the risk of intra-uterine transmission remains uncertain (Percher et al., 2016). Are transmissions in exclusively formula-fed infants due to infection at the time of delivery rather than earlier and, if so, what is the route of infection and how can this be prevented?

### Protection Against Intra-Uterine Infection

Little is known about the possible mechanisms associated with protection against intra-uterine infection. Apoptosis of infected cells may contribute to control of HTLV-1 infection. Placentas from HTLV-1 infected women presented a higher number of apoptosis-positive cells than those from non-infected women (Fujino et al., 1999). Trophoblasts were infected by HTLV-1 when co-cultured with MT-2 cells resulting in trophoblast apoptosis (Fujino et al., 1999). In another *in vitro* study, malignantly transformed trophoblast cells (choriocarcinoma) were infected by HTLV-1, however, the infection was essentially non-productive, as viral protein expression could not be detected (Liu et al., 1995). Interestingly, syncytiotrophoblast co-infection with HTLV-1 and human cytomegalovirus, resulted in a productive infection of both virus (Tóth et al., 1995). Similar results were observed with Epstein-Barrar virus co-culture (Tóth et al., 1997), raising the question about the role of co-infections in the risk of HTLV-1 MTCT. Women co-infected with Strongyloides showed higher HTLV-1 transmission rates in breast-fed children when compared to uninfected women (Gotuzzo et al., 2007).

Maternal antibodies may also play a role in protection of infection. Several studies identified anti-HTLV-1 antibodies in cord blood (CB) cells from HTLV-1 carriers. Notably none of them identified the presence of anti-HTLV IgM in cord blood, which is often considered a marker of intra-uterine infection (Tsuji et al., 1990; Hino, 2011). The presence of specific IgG, that crosses the placenta, was detect in virtually all tested CB samples (Saji et al., 1989; Tsuji et al., 1990). The strong correlation of CB anti-HTLV-1 IgG with maternal antibodies (coefficient of correlation = 0.85) and the invariable decrease at a rate of 1/10 every 2 months, indicates that they were passively transferred (Tsuji et al., 1990). Importantly, HTLV-1 seropositive cord blood plasma inhibits *in vitro* infection of neonatal lymphocyte co-cultured with breast milk cells of HTLV-1 positive mothers, pointing to the protective role of passively transferred maternal antibody (Takahashi et al., 1991).

### Risk Factors for HTLV-1 MTCT

**FIGURE 3:**
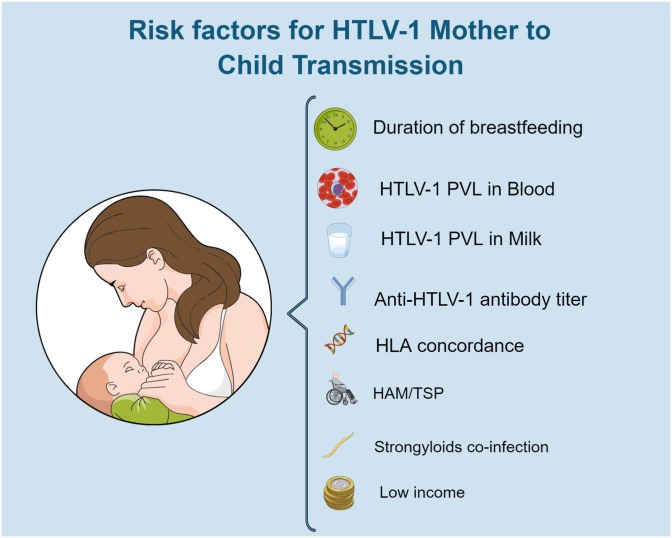
Risk factors for HTLV-1 mother to child transmission. Original figure created with mindthegraph.

Several factors are reported to influence the HTLV-1 MTCT rate (Figure 3; Nyambi et al., 1996; Ureta-Vidal et al., 1999; Hisada et al., 2002; Carles et al., 2004; Li et al., 2004; Gotuzzo et al., 2007; Kendall et al., 2009; Paiva et al., 2018). Maternal risk factors include HTLV-1 PVL in PBMCs and in breast milk (Yoshinaga et al., 1995). As patients with HTLV-1 associated diseases usually have high PVL, it seems plausible that they are at a high risk of transmitting HTLV to their offspring. In a study conducted in Peru, while 6% of the offspring of ACs were infected, the transmission rates were 19 and 31% among HAM/TSP women and those co-infected with *Strongyloides stercoralis*, respectively, even with similar duration of breastfeeding (Gotuzzo et al., 2007). This raised the hypothesis that women who are predisposed to HAM/TSP or strongyloidiasis are the same women who transmit the infection to their children (Gotuzzo et al., 2007).

### Breast Milk Cells and Transmission

The cellular mechanism of HTLV transmission through breastfeeding is not well stablished. Breast luminal epithelial cells are permissive to HTLV-1 infection, but their role and importance as a HTLV-1 reservoir *in vivo* is not clear. *In vitro*, these infected cells have altered morphology and increased ability to overcome senescence compared to non-infected cells. They can also infect epithelial cells of milk and intestinal origin as well as leukocytes (from blood and milk). Moreover, infected breast luminal epithelial cells can activate primary T cells, which is important to enhance HTLV-1 infection (Southern and Southern, 1998). Breast milk macrophages (BrMMφ) can also be infected with HTLV-1 *in vitro*. Infected cells had provirus integration in their genome, expressed high levels of intracellular HTLV-1 p24 and produced more extracellular HTLV-1 p19 than MT-2 cells. Moreover, HTLV-infected BrMMφ maintained phagocytic activity to uptake and process foreign antigen and the ability to produce co-stimulatory molecules (CD80, CD86, CD40). In the other hand, they did not express a complete DC phenotype (lack of expression of CD14 and CD11b). The transmissibility of HTLV-1 to T cells by infected BrMMφ was more efficient than from MT-2 cells. Acid medium did not alter the capacity of HTLV-1 transmission by these cells, indicating that they can transmit HTLV-1 to other cells even after exposure to acid (Takeuchi et al., 2010).

### Mechanism of Transmission Across the Gastro-Intestinal Tract

It has been proposed that a baby can ingest a total of 10^8^ HTLV-1 infected cells before weaning (Yamanouchi et al., 1985). Post-natal HTLV-1 infection must involve the migration of HTLV-1 infected cells and/or cell-free virions across the epithelium of the oro-pharynx or gastro-intestinal tract. Different mechanisms have been proposed. The infected cells could: (1) cross a disrupted epithelium; (2) cross intact epithelium (as seen with HIV infected cells); (3) attach to epithelial cells and transmit HTLV by cell-to-cell contact to epithelial cells; (4) produce cell-free HTLV-1 virions that cross the epithelium by transcytosis (Pique and Jones, 2012). *In vitro* studies have demonstrated that infected lymphocytes do not alter epithelial barrier integrity, in contrast to what is observed in the blood brain barrier. Moreover, enterocytic cell lines are not permissive for HTLV-1. The same study demonstrated that infected lymphocytes have a lower passage rate through this barrier than non-infected lymphocytes but that HTLV-1 virions can cross the tight epithelial barrier by transcytosis. After transcytosis, the virions were able to infect underlying dendritic cells (Martin-Latil et al., 2012). The importance of a viral synapse, demonstrated *ex vivo*, has been postulated for lymphocyte – lymphocyte transmission (Igakura et al., 2003). This allows targeted production of HTLV-1 virions at the point of cell-cell contact and explains the lack of cell-free virions in plasma (Demontis et al., 2015). Whether this occurs in MTCT is unknown.

### Maternal Proviral Load and the Risk of MTCT

Maternal HTLV-1 PVL is also a risk factor for HTLV-1 transmission (Ureta-Vidal et al., 1999; Hisada et al., 2002; Paiva et al., 2018). Hisada et al. considered the risk of transmission negligible when maternal PVL is lower than 0.1%. The probability of transmission increases significantly when PVL is higher than 3%. PVL in milk, which correlates with PVL in maternal PBMC (Li et al., 2004), is another risk factor for HTLV-1 transmission. PVL in breast milk was significantly higher among those women who transmitted HTLV to their children (1.3% vs. 0.18%) irrespective of duration of breastfeeding (Li et al., 2004). It has been reported that the number of HTLV-1 infected cells in breast milk is lower than in peripheral blood (Li et al., 2004), however, while the PVL in blood is usually evaluated in PBMCs, lymphocytes may not be the main cellular population observed in breast milk. In fact, when evaluating the lymphocyte population the amount of HTLV-1 infected cells in blood and breast milk is similar (Yoshinaga et al., 1995). Therefore, an alteration in the cellular composition of breast milk may influence the transmission of HTLV. In this context, conditions like mastitis may affect the risk of HTLV-1 transmission (Barcellos et al., 2005; Hisada et al., 2005), as observed in HIV infection (Semba et al., 1999). Unfortunately, there are no data on this nor on the dynamics of HTLV PVL in milk over time.

### Duration of Breastfeeding as an Important Risk Factor for MTCT

Duration of breastfeeding is one of the most important risk factors associated with HTLV-1 transmission. While short-term breastfeeding (<6 months) is associated with low transmission rates, breastfeeding for longer periods increase the risk of HTLV-1 transmission (Table 2; Takahashi et al., 1991; Oki et al., 1992; Wiktor et al., 1993, 1997; Ureta-Vidal et al., 1999; Ando et al., 2003, 2004; Paiva et al., 2018). This can be associated with different factors, such as: an increased cumulative risk of exposure to HTLV-1 present in breast milk and decrease of maternal neutralizing antibody in infants over time.

Virtually all babies born from HTLV-1 infected mothers have anti-HTLV-1 antibody at birth. These antibodies decrease exponentially in the first 3–6 months and most babies are seronegative at 6–9 months. By 12 months anti-HTLV-1 maternal antibodies are no longer detected in almost all (98.4%) of children (including those who later become infected through late breastfeeding). Among those who were infected, seroconversion occurred 6–24 months after birth with only IgG and not IgM detectable (Takahashi et al., 1991). Therefore, the risk of HTLV-1 infection increases as the duration of breastfeeding increases and the quantity of transplacental maternal antibodies decreases. These antibodies may act in conjunction with the innate immune system. Antibody dependent cellular cytotoxicity (ADCC) can control HTLV-1 infection *in vitro* in a mechanism that seems to involve NK cells (Biggar et al., 2006). Anti-HTLV-1 antibodies were not detected in 60 breast milk samples, diluted 1:10, from infected HTLV-1 mothers (Kinoshita et al., 1984, 1985, 1987).

### Breast Milk Constituents and It Influence in HTLV Transmission

Milk constituents may also interfere in HTLV-1 transmission. Lactoferrin, for example, is able to facilitate HTLV-1 replication in lymphocytes derived from ACs. It also improves HTLV transmission to CB lymphocytes. This protein can transactivate the long terminal region (LTR) promoter and, interestingly, this activation is more prominent in CB mononuclear cells than in PBMCs. In the other hand, the same protein suppressed viral entry, although to a lesser degree than was observed for HIV (Moriuchi and Moriuchi, 2001). Moriuchi and colleagues also demonstrated that prostaglandin E 2 (PGE2) and transforming growth factor β (TGF-β), that are abundant in breast milk, can transactivate LTR of HTLV-1, facilitating HTLV-1 replication of infected lymphocytes derived from ACs. Moreover, *in vitro*, both TGF-β and PGE2 were able to enhance HTLV-1 transmission to CB lymphocytes (Moriuchi et al., 2001; Moriuchi and Moriuchi, 2002). HTLV-1 tax protein was able to stimulate PGE2 expression in PBMC and CB mononuclear cells (Moriuchi et al., 2001), while TGF-β induced biding of HTLV-1 virions and expression of GLUT1 (HTLV-1 receptor) in CD4+ T lymphocyte (Jones et al., 2005).

### Other Maternal Factors That Can Influence HTLV-1 MTCT Risk

Maternal anti-HTLV-1 antibody titer has also been associated with MTCT risk. However, the association between antibody titer and risk of HTLV-1 transmission may be related to the strong correlation observed with antibody titer and PVL (Li et al., 2004). This correlation is also observed in HTLV-1 carriers, despite pregnancy (Mueller et al., 1996).

When PBMC and BMMC of HTLV-1 infected mothers are cultured they produce HTLV-1 antigen positive cells. Interestingly, 27.9% can be considered high HTLV-1 antigen producing mothers, while 72.1% are considered low antigen producing mothers. The transmission rate of HTLV-1 was 37.5% among high and 3.2% for low antigen producing mothers, independent of duration of breastfeeding. While the production observed was parallel in PBMC and BMMC from the same patient, it did not correlate with maternal anti-HTLV-1 antibody titers, indicating that the cytotoxic T cell response could be more important than humoral response to control HTLV-1 load and, consequently, vertical transmission (Yoshinaga et al., 1995).

Human Leukocyte Antigen (HLA) concordance between mothers and child is another recognized risk factor for HTLV-1 transmission. In Jamaica, HTLV-1 transmission risk increased (1.75-fold) with each increase in concordance in HLA class I types (Biggar et al., 2006). Maternal infected cells present in breastmilk seem to play an important role in vertical transmission. If these cells are HLA concordant, they are not recognized as foreign by the infant immune system, and therefore they could persist. Self HLA can also activate NK-cell inhibitor receptors and suppress the immune response. Moreover, HLA concordance can enhance cell-to-cell interaction improving HTLV-1 transmission between cells (Biggar et al., 2006).

Understanding the persistence of HTLV-1 infected maternal lymphocytes in the neonates, whether through the *in utero* transplacental or the post-partum gastro-intestinal trans-epithelial route will be important for the design of interventions, particularly post-exposure antiretroviral prophylaxis.

## Neonatal Infection and the Risk of Disease Development

What we need to know about neonatal infection

• What are the consequences of neonatal infection in early life as well as in adult life?
• Are these consequences different if infection occurred *in utero*?
• What is the relative contribution of HTLV-1 MTCT to the global burden of HTLV-1 associated disease?
• How and when should HTLV-1 infection be diagnosed in children?

### Consequences of Neonatal Infection in Early Life as Well as in Adult Life

Orally infected young rats present higher PVL in spleen cells when comparing to intraperitoneal infection. In this animal model, oral infection was associated with impaired HTLV-1 specific cellular immune response (evaluated by IFN-γ production and HTLV-1 specific cell proliferation response) and no production of HTLV-1 specific antibodies (Hasegawa et al., 2003). Therefore, oral infection, as occurs mainly in MTCT, may, through unknown mechanism, be associated with low immune response and high PVL which are both associated with risk of disease development (Kannagi et al., 2004). Ueno et al. (2012) reported that adult ACs infected by MTCT had higher PVL (2.41 copies of HTLV-1 pX gene/100 PBMC) than those infected by sexual intercourse (0.34 copies of HTLV-1 pX gene/100 PBMC), however the time from infection was not evaluated in this study (Ueno et al., 2012). This supports the hypothesis that vertical transmission can be associated with high risk of HTLV-1 associated disease onset, which could contribute to explain some high prevalence of disease among certain family members (Pombo-De-Oliveira et al., 2001; da Silva et al., 2013; Alvarez et al., 2016; Mendes et al., 2016). Exposure to HTLV-1 at the beginning of life has been associated with a higher risk of ATL (Hisada et al., 1998, 2001; Kannagi et al., 2004). HAM/TSP development can also occur following vertical transmission (Yoshida et al., 1993; Araújo et al., 2002; Kendall et al., 2009; Oliveira et al., 2018; Varandas et al., 2018).

Data on the impact of HTLV-1 on child development are scarce. HTLV-1 neonatal infection does not seem to impact the early children development, as no difference in reaching the development milestones was detected in a total of 68 HTLV-1 infected compared with 80 uninfected Peruvian children born to HTLV-1 infected mothers (Montano et al., 2004; Kendall et al., 2009). As the risk of HTLV-1 infection during short term breastfeeding is very low, it is possible that most children infected by vertical transmission are not infected at the time of these milestone evaluations.

### What Is the Impact of HTLV-1 on Routine Blood Parameters in Children?

Maloney et al. (2003) evaluated laboratorial parameters of 28 HTLV-1 infected and 280 uninfected children from Jamaica. Although the mean hemoglobin level was similar between these two groups, children with HTLV-1 seemed to have a higher rate of severe anemia (RR = 2.5/CI = 0.8–7.9) (Maloney et al., 2003). HTLV-1 seropositive children with severe anemia had higher HTLV PVL (9.6 copies/100 PBMC) compared to those without anemia (3.4 copies/100 PBMC). Social economic status may partially account for this association, since following adjustment for maternal income, this association did not achieve statistical significance (*p* = 0.06). The anemia was microcytic hypochromic, indicative of iron deficiency, which can also result from parasitic diseases and malnutrition. Both of which can be linked with poor socioeconomic status (Maloney et al., 2004). The plasma concentrations of IFN-γ, TNF-α, IL-1a, IL-4, IL-6, and IL-10 were not associated with the development of severe anemia in those children, but this could be due to the small sample size (Maloney et al., 2004). Interestingly, HTLV-1 infected children with infective dermatitis were anemic when compared to an age matched control group with atopic eczema (La Grenade et al., 1998).

HTLV-1 infected children in Jamaica also present higher rates of HLA-DR – positive CD4 T cells when compared to non-infected children (1.9% vs. 1.12%, *p* < 0.001), which strongly correlated with time since seroconversion (*r* = 0.74, *p* = 0.009) and the percentage of CD25CD4 T cells (*r* = 0.7, *p* = 0.02) (Maloney et al., 1995). Children with infective dermatitis associated with HTLV-1 (IDH) also presented higher T cell activation markers expression (HLA-DR) when compared to children with atopic eczema. They also have high percentage of CD4 and CD8 cells (La Grenade et al., 1998).

### Infective Dermatitis, ATL and HAM/TSP in Children

Infective dermatitis associated with HTLV-1 (IDH) is also observed in children infected early in infancy, and is associated with a high PVL and increased risk for the development of other diseases such as ATL and HAM/TSP (LaGrenade et al., 1990; Bittencourt et al., 2006; Hlela et al., 2013). Varandas et al. (2018) reported that 54% of patients with IDH developed HAM/TSP (Varandas et al., 2018).

Children infected with HTLV-1 report more frequently leg weakness, lumbar pain, paraesthesia/dysesthesia, urinary incontinence and constipation than those uninfected children. Clonus and lower extremity hyperreflexia were associated with HTLV-1 infection, Therefore, it is important to perform neurological evaluation of HTLV-1 infected children, once it can detect otherwise-unrecognized neurological disease (Kendall et al., 2009). HTLV-1 infected children also presented higher rates of eczema, seborrheic dermatitis, and persistent hyperreflexia of lower limbs when comparing to non-infected children in Jamaica (Maloney et al., 2003).

There are many reports of early onset of HTLV-1 associated diseases, such as HAM/TSP and ATL (Araújo et al., 2002; Bittencourt et al., 2006; Varandas et al., 2018). A review about this topic was published recently, and the authors found 27 published cases of juvenile HAM/TSP and 31 cases of ATL in infancy (Oliveira et al., 2018). Rapid progression of HAM/TSP in children has been reported (Oliveira et al., 2018; Varandas et al., 2018) but whether the rate of progression in younger patients is faster than in adults is uncertain. Among adult patients, early age at onset of clinical signs may be associated with rapid progression of HAM/TSP (Kuroda et al., 1991).

Juvenile HAM/TSP occurs predominantly in females and is frequently associated with IDH (history or co-incidence in up to 55.6%) (Oliveira et al., 2018). In Japan, in 10% of HAM/TSP patients, disease onset was before 15 years of age (Yoshida et al., 1993). Short stature and association with pseudoparathyroidism have been reported in HAM/TSP children (Yoshida et al., 1993; Araújo et al., 2002).

A descriptive study of 5 patients with juvenile HAM/TSP showed normal development among all patients, who reached the development milestone at normal age (Araújo et al., 2002). However, in a Japanese report, slight delay in psychomotor development was observed in juvenile HAM/TSP cases (Yoshida et al., 1993). The most common complaint at the beginning of clinical presentation of the five cases reported in Brazil was the inability to run as fast as children of the same age and/or pain in the legs (Araújo et al., 2002).

ATL usually occurs in adults (Iwanaga et al., 2012; Satake et al., 2015; Nosaka et al., 2017) and the onset of ATL in infants is rarely reported. Genetic mutations in protein-coding genes associated with cell cycle regulation have already been implicated as a possible predisposing factor for ATL outcome in children (Pombo-de-Oliveira et al., 2002). The small number of available samples preclude a definitive conclusion. Among those published cases compiled by Oliveira et al. (2018), 16.1% of pediatric ATL were associate with IDH, while 6.4% also had HAM/TSP. As seen in adults, the clinical presentations in children vary, with distinct prognosis. Acute, chronic and smoldering ATL have all been reported in infants. Interestingly, most cases were reported in Latin-American countries (Oliveira et al., 2018). Despite the severity of the disease, there is a paucity of information on this subject and HTLV-1 infection should be also investigated in oncologic pediatric patients.

### Are the Consequences of HTLV-1 Infection Different If the Transmission Occurred *in utero*?

There are no data on whether *in utero* infection alters HTLV-1 infection outcomes in the short or long term.

### Relative Contribution of HTLV-1 MTCT to the Global Burden of HTLV-1 Associated Disease

Until now, there is no cure to the lifelong HTLV-1 infection nor any preventive measure to avoid the onset of HTLV-1 associated diseases in patients living with HTLV-1. Thus, infection prevention is extremely important. While almost all ATL and IDH cases are associated with maternal transmission (The T and B-cell malignancy study group, 1988), MTCT can account for around 30% of HAM/TSP cases (Bartholomew et al., 1998). As early age at first sexual intercourse can increase the risk of HAM/TSP (Krämer et al., 1995), we can postulate that the infection early in life may also be linked to a higher risk of HAM/TSP. Indeed, Kaplan et al. (1990) estimated a higher cumulative lifetime risk of HAM/TSP among those individuals infected at birth (0.0023) when compared with those infected later in life (0.0018, 0.0014, and 0.008 if infected at ages 20, 30, and 50 years, respectively). These are considered conservative estimates applicable to Japan and may differ from other regions (Kaplan et al., 1990). The lifetime risk of HAM/TSP is higher in other regions, for example, 1.8% in Jamaica and Trinidad (Maloney et al., 1998) and that is a report of lifetime risk of up to 9% in Brazil (Tanajura et al., 2015). Moreover, in a mathematical model simulating the dynamic changes of HTLV-1 carries rates, the prevention of MTCT was shown to be much more effective to reduce the rates of HTLV-1 infected carriers than the prevention of horizontal transmission (Oguma, 1990). Thus, as almost all ATL and infective dermatitis cases and up to 30% of HAM/TSP cases are associated with MTCT we can conclude that MTCT contributes disproportionately more to the global burden of HTLV-1 associated diseases when comparing to others transmission routes. Therefore, antenatal screening and avoidance of breastfeeding would not only result in a decrease in HTLV-1 incidence, but most importantly a sharp decrease in the onset of HTLV-1 associated diseases. Thus, it can be considered the single best approach to reduce disease.

### How and When Should HTLV-1 Infection Be Diagnosed in Children?

The diagnosis of HTLV-1 neonatal infection can be challenging. Passively transmitted antibodies can be detected in new-borns from HTLV-1 positive mothers. These antibodies decrease gradually and at 12 months the great majority of children do not have anti-HTLV-1 maternal antibodies anymore. In the other hand, seroconversion of infected children can occur from months to more than 2 years after birth. No seroconversion after 3 years of age was reported (Kusuhara et al., 1987; Nyambi et al., 1996). Molecular methods such as PCR may be useful for the diagnosis of neonatal infection (Bittencourt et al., 2006), however, the diagnosis of HTLV-infection before 3 years of age usually does not impact the management of HTLV-1 infection in children, although this may vary by region. Moreover, there is no commercially available PCR for the diagnosis of HTLV-1 infection and the sensitivity of PCR in children should be assessed in further studies. Two distinct approaches are possible: serological – testing children born to non-breasting HTLV-1 infected mothers once at 15 months or if breastfeeding 3 months after completed weaning; molecular – which will allow early diagnosis but will necessitate multiple sampling. At present there is no consensus and research into parental attitudes and their willingness (to be screened, to use formula feeding, short-term breastfeeding, how often the children should be tested, at each age, for example) will be important to inform any guidelines.

## Prophylactic Measures to Avoid HTLV-1 Vertical Transmission

What we need to know about prophylactic measures

• Does pregnancy impair the detection of HTLV-1 infection?
• Should we recommend avoidance of breastfeeding to all HTLV-1 seropositive women?
• Cost-effectiveness analysis of HTLV-1 antenatal screening in different scenarios
• Should short-term breast feeding, and freeze and thawed milk be recommended as a prophylactic measure to MTCT?
• Safety and efficacy of antiretroviral therapy (ARV) as a prophylactic method for MTCT
• What is the role of neutralizing antibodies in the prevention of MTCT?
• What is the impact, if any, of pre-labor elective cesarean section in the prevention of MTCT?
• Approaches to a successful antenatal screening program: education of general population, doctors, pregnant women

### Detection of HTLV-1 Infection in Pregnancy

In order to consider any measure to avoid MTCT, it is essential to acknowledge HTLV infection in pregnant women. However, HTLV-1 antenatal screening is only mandatory in Japan and part of clinical practice in other areas, such as French Guiana and some Brazilian States, as Bahia.

In general population serological tests, such as ELISA, are widely recommended as a screening test, followed by confirmatory assay (usually western blot or PCR). More recently, chemiluminescent microparticle immunoassay (CMIA), electrochemiluminescent assays (ECLIA), and line immunoassays (LIA) became available for HTLV-1 diagnosis. Such tests have proven robust for the detection of other blood borne infections in the ante-natal population without loss of sensitivity. However, the National Screening Committee in the United Kingdom have cited lack of evidence that current HTLV assays detect this infection in pregnant women as one reason not to recommend the addition of HTLV-1 testing to the existing national antenatal screening programme (UK National Screening Committee, 2017).

Therefore, the questions which arise are how we should test pregnant women and when? The use of pooled samples to reduce the costs of antenatal screening can be considered (Ramos et al., 2011), but this may affect the sensitivity of the screening test (Andersson et al., 2001). Dried blood spots may also present advantages when compared to venous blood. Dried blood spots on filter paper showed 100% sensitivity, specificity and diagnostic accuracy when compared to venous blood sample (Boa-Sorte et al., 2014). In a study of almost 120,000 pregnant women donating cord blood Glasser et al. (2013) detected a total of 545 reactive samples but some degree of discordance with two screening assays.

Several studies have used different techniques in order to diagnosis HTLV-1 infection during pregnancy (Supplementary Table). Tsuji et al. (1990) did not observed significant difference in the prevalence among pregnant women and age matched blood donors (PA and IF), although a little higher in the first group (3.9% vs. 1.6%) (Tsuji et al., 1990). Beside this, the higher prevalence among pregnant women, when compared to blood donors (Taylor and The Htlv European Research Network (HERN), 1999; Taylor et al., 2001) argues against any lack of sensitivity. On the other hand, the necessity of confirmatory tests for HTLV-1 diagnosis is evident, regardless of which population is being evaluated.

In a scenario of limited resources and many demands in public health, cost-effectiveness analysis is required to guide policy makers. In Nagasaki (Japan), in the late 80’s, with 4% prevalence, the cost for detecting a HTLV-1 infected person, for preventing an infection and preventing a case of ATL was 50,000, 250,000, and 5,000,000 (JYE), respectively. At the time of the national screening program implementation in Japan (2010), considering the prevalence of 0.1% the estimated cost was 40-fold higher, according to Hino (2011). A recent study demonstrated that antenatal screening can be cost-effective in United Kingdom (Malik and Taylor, 2018). It is important to evaluate the cost-effectiveness in different scenarios.

Even if antenatal screening program is implemented in a country, success will depend on many factors including education of obstetrics, pediatrics, and general community (Hino, 2011). The majority of HTLV-1 seropositive women affirmed that they had not heard about HTLV-1, even when 72.5% were aware that they had a blood virus and only 10% of pregnant women were aware of other modes of transmission (Oni et al., 2006). The lack of information may influence the mothers to keep breastfeeding, in spite of medical recommendation, in order to maintain confidential their carrier status from family members and community (Hino, 2011). Even in high prevalence countries, such as Brazil, many doctors are not aware of HTLV-1 infection (Zihlmann et al., 2012). Many do not have any experience of ATL and believe that it is treatable and rare, which influences in their commitment to recommend against breastfeeding for HTLV-1 seropositive mothers (Hino, 2011).

### Avoidance of Breastfeeding

The avoidance of breastfeeding has conclusively been shown to be an efficient measure to prevent post-partum HTLV-1 MTCT (Ribeiro et al., 2012; Leal et al., 2015) and is the key component of the Japanese HTLV antenatal screening programme (Nishijima et al., 2019). However, the risks and benefits of formula-feeding need to be evaluated in each setting. In resource-poor settings, where alternatives to breast milk are limited and the prevalence of infectious diseases are high, poor infant outcomes related to gastroenteritis, chest infections etc. may outweigh the benefit of avoiding HTLV-1 infection. Therefore, the recommendation of avoidance of breastfeeding should take in account the conditions of the parturient and their access to methods other than breast-feeding which need to be safe, affordable, accessible, acceptable, and sustainable (Hisada et al., 2005; van Tienen et al., 2012).

In this context, it is also important to consider if the presence of risk factors should be taken into account to establish who should avoid breastfeeding. For example, the PVL in mother‘s blood or in breast milk, could be considered to guide this recommendation? The availability of adequate substitutes of breastmilk should also be considered (Hisada et al., 2005). Finally, if breast-feeding is considered the best option, can this be made safer for the infant?

Therefore, it is necessary to discuss whether the benefits of breastfeeding will outweigh or not the risk of HTLV-1 infection, and if this is applicable to all scenarios. This remains one of the main questions regarding HTLV-1 MTCT.

### Short-Term Breastfeeding

Early weaning is associated with low risk of HTLV-1 transmission (Table 2) and therefore, can be recommended to reduce the transmission, mainly in those situation when there is no or limited access to alternatives for feeding the infants (Hisada et al., 2005).

In fact, Takahashi et al. (1991) showed that the risk of infection by short-term breastfeeding is lower (4.4%) than long term breastfeeding (14.4%) (Takahashi et al., 1991). Similar results were reported by Hino et al. (1996), however, they were reluctant to affirm that short-term breastfeeding is as safe as formula-feeding. Indeed, in a large study from Japan, a higher incidence of HTLV-1 infection among short-term breastfeeding group (7.5%) than in bottle-fed infants was reported (2.5%) (Hino, 2011). Since 2016, Japan recommends only formula feed for all HTLV-1 seropositive mothers. Short-term breastfeeding and freeze-thawing breast milk are not recommended anymore (Nishijima et al., 2019). If short-term breastfeeding is adopted, how long should be the breastfeeding? The advantages and risks of an early weaning at, for example, 3 months and 6 months should be evaluated. Moreover, some mothers may have difficulties to respect early weaning and may breastfeed for longer periods than recommended, which can increase the transmission risk. Thus, this should also be considered when recommending early weaning.

### Using Freeze-Thawed Breastmilk

The process of freezing and subsequently thawing the breast milk results in morphological changes and loss of function of cells present in milk (Ando et al., 1989). Babies fed with breastmilk from infected mothers submitted to frozen and thawed process were not infected (Ando et al., 1989). However, in this study, the duration of breastfeeding was not specified. As this process can be laborious for new mothers, it could result in short-term breastfeeding, which could also contribute to the low transmission rate. In fact, in the study of Ando et al. (2004) none of 54 babies who were fed with freeze-thawed breastmilk for a short period were infected even after 12 years follow-up. However, the mean time of breastfeeding duration was 2 months (varying between 2 weeks – 6 months) (Ando et al., 2004). As discussed before, short-term breastfeeding results in a low transmission rate. In this study, the process consisted in freezing milk -12°C overnight then thawing it at 37°C in a water bath (Ando et al., 2004).

### ARV Therapy During Pregnancy and in Neonate

Breast milk and breastfeeding present numerous benefits including emotional, nutritional, cognitive and immunological benefits (Lawrence and Lawrence, 2004). So, in a context, where we must balance the benefits of breastfeeding and the risk of HTLV-1 MTCT, alternatives to avoidance of breastfeeding should be considered. In this scenario, the implementation of antiretroviral therapy (ART) could be an alternative. ART is well known as an effective measure to prevent HIV MTCT (Leal et al., 2015). Although antiretroviral drugs do not seem to be effective in controlling a stablished HTLV infection and in reducing PVL (Taylor et al., 2006), *in vitro* studies demonstrated that drugs, such as Zidovudine, Adefovir, and the prodrugs Adefovir dipivoxil and Tenofovir disoproxil can inhibit HTLV-1 infection of lymphocytes *in vitro* (Macchi et al., 1997; Hill et al., 2003) and prevent *de novo* cellular infection (Bertazzoni et al., 2018). As demonstrated *in vitro*, free infectious HTLV-1 virions can cross the human epithelial barrier via transcytosis and infect subjacent target cells such as dendritic cells (Martin-Latil et al., 2012), so, ARV could be beneficial to avoid or reduce the risk of vertical transmission. However, clinical trials should be implemented in order to assess benefits and risks of ART to prevent HTLV-1 MTCT. Moreover, analysis regarding costs and medical access to therapeutic drugs should be considered.

### Neutralizing Antibodies

The potential of passive immunization to prevent HTLV-1 infection was been demonstrated in distinct animal models (rabbit, mice, monkey) (Sawada et al., 1991; Miyoshi et al., 1992; Murata et al., 1996; Saito et al., 2014). As HTLV-1 has a conserved genome, monoclonal antibodies can be considered instead of polyclonal antibodies obtained from ACs. Human monoclonal antibodies capable of neutralizing HTLV-1 have been developed (Kuroki et al., 1992).

Weekly intraperitoneal inoculation of anti-HTLV antibodies in new-borns rabbits (from 1 week of age and up to weaning) reduced HTLV-1 transmission to rabbit offspring (42.9% vs. 8.3%) (Sawada et al., 1991). HTLV-1 neutralizing monoclonal antibody of rat origin (LAT-27) can be passively transfered from pregnant rats to their offspring. The transferred antibodies were able to neutralize HTLV-1 *in vitro* and protected the infants against intra-peritoneum HTLV-1 infection. Moreover, similar results were observed when using humanized LAT-27 antibodies in humanized immunodeficient mice (NOG mice). Both *in vitro* neutralization of HTLV-1 and *in vivo* resistance for HTLV-1 infection were acquired (Fujii et al., 2016). Unfortunately, this antibody was not able to inhibit oral HTLV-1 infection in rats, despite the protection of intraperitoneal infection (Murakami et al., 2017). This reinforces the importance of understanding the cellular mechanism of HTLV-1 transmission in order to develop preventive measures to avoid HTLV-1 vertical transmission.

### Pre-labor Elective Cesarean Section

A study conducted in 1995 evaluated the presence of proviral DNA in maternal blood at delivery, cord blood and oral aspirates from babies delivered by vaginal route and cesarean section. They found that all maternal bloods were positive while all cord bloods were negative. Interestingly, 4/10 new-borns from vaginal route had HTLV-1 proviral DNA in oral aspirates while only one out of seven born by cesarean section had, indicating HTLV exposure during passage through the birth canal (Sawada et al., 1995).

In a cohort of 41 HTLV-1 infected pregnant women in Brazil, 81% delivered by elective cesarean section and all babies were bottle-feed. None of those children were HTLV-1 positive when tested by PCR from 3 to 39 months. The authors hypothesized that the delivery method, together with the avoidance of breastfeeding, contributed to the prevention of vertical transmission (Bittencourt et al., 2002).

In Okinawa (Japan) a decrease in the transmission rate among non-breastfeed children was observed between 1986–1991 (12.8%) and 1995–1999 (3.2%). Although the difference was not statically significant, the authors highlighted that since 1991, doctors have been advised to protect new-borns against HTLV-1 nosocomial infection at delivery. Therefore, obstetricians and gynecologists have taken precautions to avoid new-borns contamination at the time of delivery, ensuring that they do not swallow blood during birth. They hypothesize that this action contributed to the observed decrease (Kashiwagi et al., 2004). More studies are clearly needed in order to evaluate the impact of pre-labor elective cesarean section in the prevention of vertical transmission of HTLV-1.

## Final Considerations

**FIGURE 4:**
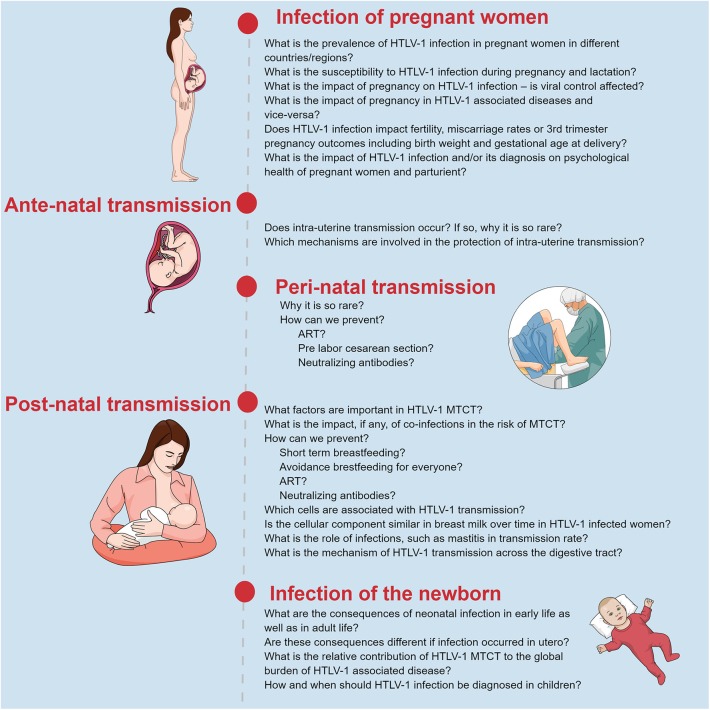
Summary of unmet questions regarding HTLV-1 mother to child transmission. Original figure created with mindthegraph.

Soon after the discovery of HTLV-1, MTCT has been implicated as one of the most important modes for HTLV transmission and for maintaining HTLV-1 within a population. However, little has been done in the world to avoid HTLV transmission from mother to child. Despite the success observed, Japan remains the only country to include HTLV-1 antenatal screening in their national programme. Therefore, although many research questions remain (Figure 4), the most important question is why has so little been done to prevent HTLV-1 MTCT thus far?

## Author Contributions

CR and GT contributed to conception and design of the study. CR wrote the first draft of the manuscript. All authors contributed to manuscript revision, read, and approved the submitted version.

## Conflict of Interest Statement

The authors declare that the research was conducted in the absence of any commercial or financial relationships that could be construed as a potential conflict of interest.
