# Supplementary material for: Mother-to-Child HTLV-1 Transmission: Unmet Research Needs
Source: Front Microbiol. 2019 May 8;10:999. doi: 10.3389/fmicb.2019.00999 (PMC6517543; doi:10.3389/fmicb.2019.00999)
Supplement: Supplementary file 1 [file Data_Sheet_1.PDF]

Supplementary Table

Supplementary Table. Prevalence of HTLV-1 in pregnant women, parturient, women at reproductive age and babies.

Reference	Place	Sample	Tests	Prevalence
**Europe**				
Taylor et al., 2005	Europe	234,078 pregnant women	ELISA; PCR	
		Belgium 5,000		0.08%
		France 10,398		0.10%
		Germany 58,747		0.01%
		Italy 6,000		0.03%
		Portugal 7,557		0.01%
		Spain 20,366		0.06%
		UK 126,010		0.05%
Ades et al., 2000	England	126,010 babies	PA; WB	0.05%
Nightingale et al., 1993	England (Birminghan)	3,522 pregnant women	PA; ELISA; IF; RIA; WB	0.14%
Trevino et al., 2011	Spain (Foreign women)	3,337 pregnant women	ELISA; WB or PCR	0.20%
Ramos et al., 2011	Spain (Immigrant women)	1,436 pregnant women	ELISA (pool); ELISA and WB	0.07%
Trevino et al., 2009	Spain	20,518 pregnant women (18,266 natives)	ELISA; WB and PCR	0.01%
Machuca et al.,2000	Spain	20,366 pregnant women	ELISA; WB; PCR	0.01%
Poljak et al., 1998	Slovenia	10,369 pregnant women	PA; WB	0.01%
**South America**				
Berini et al., 2013	Argentina (Different locations)	2,403 pregnant women	ELISA/ WB and nested-PCR	0.25%
Trenchi et al., 2007	Argentina (Cordoba)	3143 pregnant women	PA; IF and WB	0.19%
Guerra et al., 2018	Brazil (Pará)	324 pregnant adolescents	ELISA; PCR	0.60%
Mata et al., 2018	Brazil (Amapá)	216 pregnant , 72 breastfeeding women	ELISA; ELISA; WB	0.00%
Medeiros et al., 2018	Brazil (Paraná)	643 high risk pregnant women	CMIA; PCR	0.30%
Moura et al., 2015	Brazil (Alagoas)	54813 pregnant women	ELISA; WB	0.20%
Monteiro et al., 2014	Brazil (Rio de Janeiro)	1,204 pregnant women	CMIA; WB	0.66%
Boa Sorte et al., 2014	Brazil (Bahia)	692 pregnant women	ELISA	0.14%
Mello et al., 2014	Brazil (Bahia)	2,766 pregnant women	ELISA; WB and PCR	1.05%
Sequeira et al., 2012	Brazil (Pará)	13,382 pregnant women	ELISA; WB	0.30%
Souza et al., 2011	Brazil (Maranhão)	2,044 pregnant women	ELISA; WB and PCR	0.30%
Machado Filho et al., 2010	Brazil (Amazonas)	674 pregnant women	ELISA; PCR	0%
Ydy et al., 2009	Brazil (Mato Grosso)	2,965 puerperas	ELISA (2x); WB	0.24%
Lima & Viana, 2009	Brazil (Espírto Santo)	534 postpartum and pregnant women	ELISA	1.30%
		332 postpartum		1.70%
		202 preganant women		0.60%
Dal Fabro et al., 2008	Brazil (Mato Grosso do Sul)	116,689 pregnant women	ELISA; WB and PCR	0.10%
Magalhães et al., 2008	Brazil (Bahia)	408 pregnant women	ELISA; WB; PCR	0.98%
Pimenta et al., 2008	Brazil (Paraíba)	1,033 nursing mothers	ELISA; PCR	0.19%
Figueiró-Filho et al., 2007	Brazil (Mato Grosso do Sul)	32,512 pregnant women	ELISA; PCR	0.10%
Oliveira et al., 2006	Brazil (Goiás)	15,485 pregnant women	ELISA; PCR	0.10%
Neto et al., 2004	Brazil (São Paulo)	913 pregnant women	ELISA; WB	0.10%
Bittencourt et al., 2001	Brazil (Bahia)	6,754 pregnant women	ELISA; WB; PCR	0.84%
Broutet et al., 1996	Brazil (Ceará)	814 pregnant women	ELISA; WB	0.12%
Santos et al., 1995	Brazil (Bahia)	1,024 pregnant women	ELISA; WB	0.88%

Carles et al., 2004	French Guyana	1,716 pregnant women	ELISA; WB	4.40%
Tortevoye et al., 2000	French Guyana	3,834 pregnant women	ELISA; WB	3.80%
Tuppin et al., 1995	French Guyana			
Alarcon et al., 2006	Peru (Lima)	12,436 pregnant women	ELISA (pool); ELISA and WB	1.70%
Sanchez-Palacios et al., 2003	Peru (Huanta, El Carmen, Lima)	568 healthy women reproductive age	ELISA (2x); IF, WB and PCR	2.50%
	Pery (Huanta)			1.30%
	Peru (El Carmen)			3.80%
	Peru (Lima)			3.80%
Zurita et al., 1997	Peru	211 pregnant women	ELISA; WB	2.30%
		276 females (15-49y)		5%
**Central America**				
Wiktor et al., 1993	Jamaica	2329 pregnant women	ELISA, WB	3.50%
Maloney et al., 2006	Jamaica	9,226 pregnant women	ELISA; WB	3.80%
Maloney et al., 2003	Jamaica	9430 pregnant women	ELISA; WB	3.70%
Denis et al., 1988	Martinique	716 pregnant women	IF; ELISA; WB	2.34%
Mansuy et al.,1999	Martinique	467 pregnant women	ELISA; WB	1.93%
Tortevoye et al., 2005	Haiti	287 pregnant women	ELISA; WB	4.20%
Allain et al., 1992	Haiti	500 pregnant women	ELISA; WB or RIPA	2.20%
**North America**				
Cohen et al., 2010	United States of America	1,091 Potential human milk donors	Non specified screening test	0.55%
**Africa**				
Denis et al., 1988	Sub-Saharian Africa	814 (Ivory Coast); 63 (Mali); 61 (Niger); 281 (Senegal); 565 (Togo)	IF; ELISA; WB	1.34%
Collenberg et al., 2006	Burkina Faso	492 pregnant women	ELISA; WB	1%
Tuppin et al., 1996	Congo	2,070 pregnant women	ELISA; WB	0.70%
Apea-Kubi et al., 2006	Ghana (Accra)	517 pregnant women	PA	2.70%
Armah et al., 2006	Ghana (Accra)	960 preegnant women	PA, WB	2.10%
Moukandja et al., 2017	Gabon (Franceville)	973 pregnant women	ELISA	2.88%
Vardier et al., 1989	Côte d'Ivoire	513 pregnant women	IF; WB	1.90%
Etenna et al., 2008	Gabon	907 pregnant women	ELISA; WB and PCR	2.10%
	Gabon (Franceville)			5%
	Gabon (Libreville)			1%
	Gabon (Port Gentil)			1.20%
	Gabon (Lambaréné)			2.10%
	Gabon (Oyem)			0%
Mistro et al.,	Gambia	909 mothers	ELISA, WB	1%
Andersson et al., 1997	Guinea Bissau	1,231 pregnant women	ELISA; WB	2.30%
Udeze et al., 2018	Nigeria	276 pregnant women	ELISA	1.10%
Opaleye et al., 2015	Nigeria (Osogbo)	182 pregnant women	ELISA	24.20%
Olaleye et al., 1995	Nigeria	364 pregant women	ELISA, WB	5.50%
Fox et al., 2016	Malawi	418 mothers	ELISA; WB, if indeterminate PCR	2.60%
		534 children		2.20%
Forbi et al., 2007	Nigeria (South Western)	120 pregnant women	ELISA	16.70%

Ramos et al., 2011	Ethiopia (Southern)	556 pregnant women	CMIA	0%
Goubau et al., 1993	South Africa and Zaire (Basankusu and Gemena)	428 pregnant women South Africa	ELISA; IF; WB and dot blot	0.20%
		74 pregnant women (Basankusu; Zaire)		14.80%
		340 pregnant women (Gemena; Zaire)		2.60%
Delaporte et al., 1995	Zaire	1,166 pregnant women	ELISA; WB	3.70%
Olaleye et al., 1999	Nigeria (South-Western)	460 mothers	ELISA; ELISA	4.30%
**Asia**				
Ramalingam et al., 2001	India (Vellore)	201 pregnant women	PA; PCR	0%
Hamedi et al.,	Iran (Northeast)	407 parturient	ELISA; PCR	1.50%
Kusuhara et al., 1987	Japan	311 mothers	ELISA; IF	20.90%
Hino et al., 1985	Japan (Nagasaki)	5015 pregnant women	IF	3.70%
Tsuji et al., 1990	Japan (Nagasaki)	18,320 pregnant women	PA / IF	3.90%
Ando et al., 1993	Japan	4,659 pregnant women	IF	6.10%
Maehama, 2004	Japan (Okinawa)	17,207 preganant women	ELISA or PA; WB or IF	3.90%
Kashiwagi et al., 2004	Japan (Okinawa)	3,837 pregnant women	PA; ELISA; WB	5.6% (1989-1992)
				3.7% (1997-2000)
Goto et al.; 1997B	Japan (Kanto)	2,683 pregnant women	PA; WB	0.60%
Nakano et al., 1984	Japan (Okinawa)	1,269 pregnant women	IF	6.70%
Saji et al., 1989	Japan (Osaka)	2,192 pregnant women	ELISA; WB	1.00%
Ando et al., 1987	Japan (Okinawa)	2,232 pregnant women	IF	6.80%
Kinoshita et al., 1987	Japan (Nagasaki)	5,015 pregnant women	IF	3.70%
Yamada et al., 2014	Japan (Okkaido)	33,617 pregnant women	PA or CMIA; WB	0.10%
Suzuki et al., 2014	Japan (National)	707,711 pregnant women	ELISA or PA; WB or PCR	0.14%

PA: Particle aglutination; ELISA: enzyme-linked immunosorbent assay; WB: Western Blot; IF: Immunofluorescence; PCR: Polymerase chain reaction; CMIA: chemiluminescence microparticle immunoassay; RIA: Radioimmunoassy; RIPA: radioimmunoprecipitation

(Alarcón et al., 2006; Allain et al., 1992; Andersson et al., 1997; Ando et al., 1987, 1993; Apea-Kubi et al., 2006; Armah et al., 2006; Berini et al., 2013; Bittencourt et al., 2001; Boa-Sorte et al., 2014; Broutet et al., 1996; Carles et al., 2004; Cohen et al., 2010; Collenberg et al., 2006; Dal Fabbro et al., 2008; Del Mistro et al., 1994; Delaporte et al., 1995; Denis et al., 1988; dos Santos et al.; Etenna et al., 2008; Figueiró-Filho et al., 2007; Forbi and Odetunde; Fox et al., 2016; Goto et al., 1997; Goubau et al., 1993; Guerra et al., 2018; Hamedi et al., 2012; Hino et al., 1985; Kashiwagi et al., 2004; Kinoshita et al., 1987; Kusuhara et al., 1987; Lima and Viana, 2009; Machado Filho et al., 2010; Machuca et al., 2000; Maehama, 2004; Magalhães et al., 2008; Maloney et al., 2003, 2006; Mansuy et al., 1999; Mata et al., 2018; Medeiros et al., 2018; Mello et al., 2014; Monteiro et al., 2014; Moura et al., 2015; Nightingale et al., 1993; Olaleye et al., 1995, 1999; Olbrich Neto and Meira; Oliveira and Avelino, 2006; Opaleye et al., 2016; Pegha Moukandja et al., 2017; Pimenta et al., 2008; Poljak et al., 1998; Ramalingam et al., 2001; Ramos et al., 2011; Saji et al., 1989; Sanchez-Palacios et al., 2003; Sequeira et al., 2012; Souza et al., 2012; Suzuki et al., 2014; Taylor et al., 2005; Tortevoye et al., 2000, 2005; Trenchi et al., 2007; Treviño et al., 2009, 2011; Tsuji et al., 1990; Tuppin et al., 1995, 1996; Udeze et al., 2018; Verdier et al., 1989; Wiktor SZ, Pate EJ, Murphy EL, Palker TJ, Champegnie E, Ramlal A, Cranston B, Hanchard B et al., 1993; Yamada et al., 2014; Ydy et al., 2009; Zurita et al., 1997)

References:

Alarcón, J. O., Friedman, H. B., Montano, S. M., Zunt, J. R., Holmes, K. K., and Quinnan, G. V

(2006). High endemicity of human T-cell lymphotropic virus type 1 among pregnant women in peru. *J. Acquir. Immune Defic. Syndr.* 42, 604–9. doi:10.1097/01.qai.0000221680.52563.d5.

Allain, J. P., Hodges, W., Einstein, M. H., Geisler, J., Neilly, C., Delaney, S., et al. (1992). Antibody to HIV-1, HTLV-I, and HCV in three populations of rural Haitians. *J. Acquir. Immune Defic. Syndr.* 5, 1230–6. Available at: http://www.ncbi.nlm.nih.gov/pubmed/1333530 [Accessed January 9, 2019].

Andersson, S., Dias, F., Mendez, P. J., Rodrigues, A., and Biberfeld, G. (1997). HTLV-I and -II infections in a nationwide survey of pregnant women in Guinea-Bissau, West Africa. *J. Acquir. Immune Defic. Syndr. Hum. Retrovirol.* 15, 320–2. Available at: http://www.ncbi.nlm.nih.gov/pubmed/9292595 [Accessed December 10, 2018].

Ando, Y., Nakano, S., Saito, K., Shimamoto, I., Ichijo, M., Toyama, T., et al. (1987). Transmission of adult T-cell leukemia retrovirus (HTLV-I) from mother to child: comparison of bottle- with breast-fed babies. *Jpn. J. Cancer Res.* 78, 322–4. Available at: http://www.ncbi.nlm.nih.gov/pubmed/2884205 [Accessed January 28, 2019].

Ando, Y., Tanigawa, T., Ekuni, Y., Ichijo, M., and Tohyama, T. (1993). Family study of women showing development of antibody to human T-cell leukemia virus I and assessment of the risk of vertical transmission of the virus to their children. *J. Infect.* 27, 151–5. Available at: http://www.ncbi.nlm.nih.gov/pubmed/8228296 [Accessed January 28, 2019].

Apea-Kubi, K. A., Yamaguchi, S., Sakyi, B., and Ofori-Adjei, D. (2006). HTLV-1 and other viral sexually transmitted infections in antenatal and gynaecological patients in Ghana. *West Afr. J. Med.* 25, 17–21. doi:10.4314/wajm.v25i1.28239.

Armah, H. B., Narter-Olaga, E. G., Adjei, A. A., Asomaning, K., Gyasi, R. K., and Tettey, Y. (2006). Seroprevalence of human T-cell lymphotropic virus type I among pregnant women in Accra, Ghana. *J. Med. Microbiol.* 55, 765–770. doi:10.1099/jmm.0.46426-0.

Berini, C. A., Delfino, C., Torres, O., García, G., Espejo, R., Pianciola, L., et al. (2013). HTLV-1 cosmopolitan and HTLV-2 subtype b among pregnant women of non-endemic areas of Argentina. *Sex. Transm. Infect.* 89, 333–335. doi:10.1136/sextrans-2012-050594.

Bittencourt, A. L., Dourado, I., Filho, P. B., Santos, M., Valadão, E., Alcantara, L. C., et al. (2001). Human T-cell lymphotropic virus type 1 infection among pregnant women in northeastern Brazil. *J. Acquir. Immune Defic. Syndr.* 26, 490–4. Available at: http://www.ncbi.nlm.nih.gov/pubmed/11391171.

Boa-Sorte, N., Purificação, A., Amorim, T., Assunção, L., Reis, A., and Galvão-Castro, B. (2014). Dried blood spot testing for the antenatal screening of HTLV, HIV, syphilis, toxoplasmosis and hepatitis B and C: Prevalence, accuracy and operational aspects. *Brazilian J. Infect. Dis.* 18, 618–624. doi:10.1016/j.bjid.2014.05.009.

Broutet, N., de Queiroz Sousa, A., Basilio, F. P., Sa, H. L., Simon, F., and Dabis, F. (1996). Prevalence of HIV-1, HIV-2 and HTLV antibody, in Fortaleza, Ceara, Brazil, 1993-1994. *Int. J. STD AIDS* 7, 365–369. doi:10.1258/0956462961918103.

Carles, G., Tortevoye, P., Tuppin, P., Ureta-Vidal, A., Peneau, C., El Guindi, W., et al. (2004). [HTLV1 infection and pregnancy]. *J. Gynecol. Obstet. Biol. Reprod. (Paris).* 33, 14–20. Available at: http://www.ncbi.nlm.nih.gov/pubmed/14968050 [Accessed January 28, 2019].

Cohen, R. S., Xiong, S. C., and Sakamoto, P. (2010). Retrospective review of serological testing of potential human milk donors. *Arch. Dis. Child. Fetal Neonatal Ed.* 95, 2008–2010. doi:10.1136/adc.2008.156471.

Collenberg, E., Ouedraogo, T., Ganamé, J., Fickenscher, H., Kynast-Wolf, G., Becher, H., et al. (2006). Seroprevalence of six different viruses among pregnant women and blood donors in rural and urban Burkina Faso: A comparative analysis. *J. Med. Virol.* 78, 683–92. doi:10.1002/jmv.20593.

Dal Fabbro, M. M. F. J., Cunha, R. V. da, Bóia, M. N., Portela, P., Botelho, C. A., Freitas, G. M. B. de, et al. (2008). Infecção pelo HTLV 1/2: atuação no pré-natal como estratégia de controle da doença no Estado de Mato Grosso do Sul. *Rev. Soc. Bras. Med. Trop.* 41, 148–151. doi:10.1590/S0037-86822008000200003.

Del Mistro, A., Chotard, J., Hall, A. J., Fortuin, M., Whittle, H., De Rossi, A., et al. (1994). HTLV-I/II seroprevalence in The Gambia: a study of mother-child pairs. *AIDS Res. Hum. Retroviruses* 10, 617–20. doi:10.1089/aid.1994.10.617.

Delaporte, E., Buvé, A., Nzila, N., Goeman, J., Dazza, M. C., Henzel, D., et al. (1995). HTLV-I infection among prostitutes and pregnant women in Kinshasa, Zaïre: how important is high-risk sexual behavior? *J. Acquir. Immune Defic. Syndr. Hum. Retrovirol.* 8, 511–5. Available at: http://www.ncbi.nlm.nih.gov/pubmed/7697449 [Accessed January 7, 2019].

Denis, F., Verdier, M., Chout, R., Ramiandrisoa, H., Sangare, A., Prince-David, M., et al. (1988). [Prevalence of HTLV-1 virus in pregnant women in Black Africa, Martinique, and foreigners living in France]. *Bull. Acad. Natl. Med.* 172, 717–22. Available at: http://www.ncbi.nlm.nih.gov/pubmed/3056585 [Accessed January 9, 2019].

dos Santos, J. I., Lopes, M. A., Deliège-Vasconcelos, E., Couto-Fernandez, J. C., Patel, B. N., Barreto, M. L., et al. Seroprevalence of HIV, HTLV-I/II and other perinatally-transmitted pathogens in Salvador, Bahia. *Rev. Inst. Med. Trop. Sao Paulo* 37, 343–8. Available at: http://www.ncbi.nlm.nih.gov/pubmed/8599064 [Accessed July 10, 2017].

Etenna, S. L.-D., Caron, M., Besson, G., Makuwa, M., Gessain, A., Mahe, A., et al. (2008). New Insights into Prevalence, Genetic Diversity, and Proviral Load of Human T-Cell Leukemia Virus Types 1 and 2 in Pregnant Women in Gabon in Equatorial Central Africa. *J. Clin. Microbiol.* 46, 3607–3614. doi:10.1128/JCM.01249-08.

Figueiró-Filho, E. A., Senefonte, F. R. de A., Lopes, A. H. A., Morais, O. O. de, Souza Júnior, V. G., Maia, T. L., et al. (2007). Freqüência das infecções pelo HIV-1, rubéola, sífilis, toxoplasmose, citomegalovírus, herpes simples, hepatite B, hepatite C, doença de Chagas e HTLV I/II em gestantes, do Estado de Mato Grosso do Sul. *Rev. Soc. Bras. Med. Trop.* 40, 181–187. doi:10.1590/S0037-86822007000200007.

Forbi, J. C., and Odetunde, A. B. Human T-cell lymphotropic virus in a population of pregnant women and commercial sex workers in South Western Nigeria. 129–132.

Fox, J. M., Mutalima, N., Molyneux, E., Carpenter, L. M., Taylor, G. P., Bland, M., et al. (2016). Seroprevalence of HTLV-1 and HTLV-2 amongst mothers and children in Malawi within the context of a systematic review and meta-analysis of HTLV seroprevalence in Africa. *Trop. Med. Int. Heal.* 21, 312–324. doi:10.1111/tmi.12659.

Goto, K., Sato, K., Kurita, M., Masuhara, N., Iijima, Y., Saeki, K., et al. (1997). Serologic survey for HTLV-I in Kanagawa Prefecture. *Tokai J. Exp. Clin. Med.* 22, 7–8. Available at: http://www.ncbi.nlm.nih.gov/pubmed/9608625 [Accessed January 7, 2019].

Goubau, P., Desmyter, J., Swanson, P., Reynders, M., Shih, J., Surmont, I., et al. (1993). Detection of HTLV-I and HTLV-II infection in Africans using type-specific envelope peptides. *J. Med. Virol.* 39, 28–32. Available at: http://www.ncbi.nlm.nih.gov/pubmed/8093712 [Accessed December 10, 2018].

Guerra, A. B., Siravenha, L. Q., Laurentino, R. V., Feitosa, R. N. M., Azevedo, V. N., Vallinoto, A. C. R., et al. (2018). Seroprevalence of HIV, HTLV, CMV, HBV and rubella virus infections in pregnant adolescents who received care in the city of Belém, Pará, Northern Brazil. *BMC Pregnancy Childbirth* 18, 1–7. doi:10.1186/s12884-018-1753-x.

Hamedi, A., Akhlaghi, F., Meshkat, Z., Sezavar, M., Nomani, H., and Meshkat, M. (2012). The Prevalence of Human T-Cell lymphotropic Virus Type 1 in Pregnant Women and Their Newborns. *ISRN Obstet. Gynecol.* 2012, 975135. doi:10.5402/2012/975135.

Hino, S., Yamaguchi, K., Katamine, S., Sugiyama, H., Amagasaki, T., Kinoshita, K., et al. (1985). Mother-to-child transmission of human T-cell leukemia virus type-I. *Jpn. J. Cancer Res.* 76, 474–80. Available at: http://www.ncbi.nlm.nih.gov/pubmed/2991060 [Accessed January 7, 2019].

Kashiwagi, K., Furusyo, N., Nakashima, H., Kubo, N., Kinukawa, N., Kashiwagi, S., et al. (2004). A decrease in mother-to-child transmission of human T lymphotropic virus type I (HTLV-I) in Okinawa, Japan. *Am. J. Trop. Med. Hyg.* 70, 158–163.

Kinoshita, K., Amagasaki, T., Hino, S., Doi, H., Yamanouchi, K., Ban, N., et al. (1987). Milk-borne transmission of HTLV-I from carrier mothers to their children. *Jpn. J. Cancer Res.* 78, 674–80. Available at: http://www.ncbi.nlm.nih.gov/pubmed/2887539.

Kusuhara, K., Sonoda, S., Kazuo, T., Tokugawa, K., Fukushige, J., and Ueda, K. (1987). Mother-to-child transmission of Human T-cell leukemia virus type I (HTLV-1): A fifteen-year follow-up study in Okinawa, Japan. *Int. J. Cancer* 40, 755–757.

Lima, L. H. M. De, and Viana, M. C. (2009). Prevalence and risk factors for HIV, syphilis, hepatitis B, hepatitis C, and HTLV-I/II infection in low-income postpartum and pregnant women in Greater Metropolitan Vitória, Espírito Santo State, Brazil. *Cad. saude publica / Minist. da Saude, Fund. Oswaldo Cruz, Esc. Nac. Saude Publica* 25, 668–676. doi:10.1590/S0102-311X2009000300021.

Machado Filho, A. C., Sardinha, J. F. J., Ponte, R. L., Costa, E. P. da, da Silva, S. S., and Martinez-Espinosa, F. E. (2010). Prevalence of infection for HIV, HTLV, HBV and of syphilis and chlamydia in pregnant women in a tertiary health unit in the western Brazilian Amazon region. *Rev. Bras. Ginecol. e Obs.* 32, 176–183. doi:10.1590/s0100-72032010000400005.

Machuca, A., Tuset, C., Soriano, V., Caballero, E., and Aguilera, A. (2000). Prevalence of HTLV infection in pregnant women in Spain. Available at: www.sextransinf.com [Accessed December 10, 2018].

Maehama, T. (2004). Human T cell leukemia virus-1 in pregnancy. *Int. J. Gynecol. Obstet.* 87, 247–248. doi:10.1016/j.ijgo.2004.07.024.

Magalhães, T., Mota-Miranda, A. C., Alcantara, L. C. J., Olavarria, V., Galvão-Castro, B., and Rios-Grassi, M. F. (2008). Phylogenetic and molecular analysis of HTLV-1 isolates from a medium sized town in Northern of Brazil: Tracing a common origin of the virus from the most endemic city in the country. *J. Med. Virol.* 80, 2040–2045. doi:10.1002/jmv.21278.

Maloney, E. M., Wiktor, S. Z., Palmer, P., Cranston, B., Pate, E. J., Cohn, S., et al. (2003). A cohort study of health effects of human T-cell lymphotropic virus type I infection in Jamaican children. *Pediatrics* 112, e136-42. Available at: http://www.ncbi.nlm.nih.gov/pubmed/12897319 [Accessed January 7, 2019].

Maloney, E. M., Yamano, Y., VanVeldhuisen, P. C., Sawada, T., Kim, N., Cranston, B., et al. (2006). Natural History of Viral Markers in Children Infected with Human T Lymphotropic Virus Type I in Jamaica. *J. Infect. Dis.* 194, 552–560. doi:10.1086/506365.

Mansuy, J. M., Schlegel, L., Villeneuve, L., Mengelle, C., and Magnaval, J. F. (1999). Seroprevalence of retroviral infections among pregnant women in Martinique (French West Indies). *Am. J. Trop. Med. Hyg.* 61, 598–9. Available at: http://www.ncbi.nlm.nih.gov/pubmed/10548294 [Accessed January 7, 2019].

Mata, E. C., Bezerra, R. M., Proietti Júnior, A. A., Pamplona, L. K., Gomes, L. O., Corrêa, V. C., et al. (2018). HTLV-1/2 prevalence in two Amazonian communities. *J. virus Erad.* 4, 174–178. Available at: http://www.ncbi.nlm.nih.gov/pubmed/30050680 [Accessed January 8, 2019].

Medeiros, A. C. M., Vidal, L. R. R., Von Linsingen, R., Ferin, A. N., Bessani Strapasson, T., de Almeida, S. M., et al. (2018). Confirmatory molecular method for HTLV-1/2 infection in high-risk pregnant women. *J. Med. Virol.* 90, 998–1001. doi:10.1002/jmv.25014.

Mello, M. A. G., da Conceição, A. F., Sousa, S. M. B., Alcântara, L. C., Marin, L. J., Regina da Silva Raiol, M., et al. (2014). HTLV-1 in pregnant women from the Southern Bahia, Brazil: a neglected condition despite the high prevalence. *Virol. J.* 11, 28. doi:10.1186/1743-422X-11-28.

Monteiro, D. L. M., Taquette, S. R., Sodré Barmpas, D. B., Rodrigues, N. C. P., Teixeira, S. A. M., Villela, L. H. C., et al. (2014). Prevalence of HTLV-1/2 in pregnant women living in the metropolitan area of Rio de Janeiro. *PLoS Negl. Trop. Dis.* 8, e3146. doi:10.1371/journal.pntd.0003146.

Moura, A. A., Mello, M. J. G. de, and Correia, J. B. (2015). Prevalence of syphilis, human

immunodeficiency virus, hepatitis B virus, and human T-lymphotropic virus infections and coinfections during prenatal screening in an urban Northeastern Brazilian population. *Int. J. Infect. Dis.* 39, 10–15. doi:10.1016/j.ijid.2015.07.022.

Nightingale, S., Orton, D., Ratcliffe, D., Skidmore, S., Tosswill, J., and Desselberger, U. (1993). Antenatal survey for the seroprevalence of HTLV-1 infections in the West Midlands, England. *Epidemiol. Infect.* 110, 379–87. Available at: http://www.ncbi.nlm.nih.gov/pubmed/8472781 [Accessed January 23, 2019].

Olaleye, D. O., Ekweozor, C. C., Sheng, Z., and Rasheed, S. (1995). Evidence of serological cross-reactivities with human immunodeficiency virus types 1 and 2 and human T-lymphotropic virus types I and II in sera of pregnant women in Ibadan, Nigeria. *Int. J. Epidemiol.* 24, 198–203. Available at: http://www.ncbi.nlm.nih.gov/pubmed/7797344 [Accessed January 9, 2019].

Olaleye, D. O., Omotade, O. O., Sheng, Z., Adeyemo, A. A., and Odaibo, G. N. (1999). Human T-Cell Lymphotropic Virus Types I and II Infections in Mother – Child Pairs in Nigeria. 45.

Olbrich Neto, J., and Meira, D. A. [Soroprevalence of HTLV-I/II, HIV, siphylis and toxoplasmosis among pregnant women seen at Botucatu - São Paulo - Brazil: risk factors for HTLV-I/II infection]. *Rev. Soc. Bras. Med. Trop.* 37, 28–32. Available at: http://www.ncbi.nlm.nih.gov/pubmed/15042179 [Accessed August 21, 2017].

Oliveira, S. R. de, and Avelino, M. M. (2006). Soroprevalência do vírus linfotrópico-T humano tipo I entre gestantes em Goiânia, GO, Brasil. *Rev. Bras. Ginecol. e Obs.* 28, 467–472. doi:10.1590/S0100-72032006000800005.

Opaleye, O. O., Igboama, M. C., Ojo, J. A., and Odewale, G. (2016). Seroprevalence of HIV, HBV, HCV, and HTLV among Pregnant Women in Southwestern Nigeria. *J. Immunoass. Immunochem.* 37, 29–42. doi:10.1080/15321819.2015.1040160.

Pegha Moukandja, I., Ngoungou, E. B., Lemamy, G. J., Bisvigou, U., Gessain, A., Toure Ndouo, F. S., et al. (2017). Non-malarial infectious diseases of antenatal care in pregnant women in Franceville, Gabon. *BMC Pregnancy Childbirth* 17, 1–10. doi:10.1186/s12884-017-1362-0.

Pimenta, F. C. F., Kashima Haddad, S., de Medeiros, J. G., Costa, M. J. C., Diniz, M. F. M., Fernandes, M. P., et al. (2008). Prevalence ratio of HTLV-1 in nursing mothers from the state of Paraíba, Northeastern Brazil. *J. Hum. Lact.* 24, 289–292. doi:10.1177/0890334408316084.

Poljak, M., Bednarik, J., Rednak, K., Seme, K., Kristancic, L., and Celan-Lucu, B. (1998). Seroprevalence of human T cell leukaemia/lymphoma virus type I (HTLV-I) in pregnant women, patients attending venereological outpatient services and intravenous drug users from Slovenia. *Folia Biol. (Praha).* 44, 23–5. Available at: http://www.ncbi.nlm.nih.gov/pubmed/10730871 [Accessed January 7, 2019].

Ramalingam, S., Kannangai, R., Prakash, K. J., Ajithkumar, K., Jacob, M., George, R., et al. (2001). A pilot study of HTLV-I infection in high-risk individuals & their family members from India. *Indian J. Med. Res.* 113, 201–9. Available at: http://www.ncbi.nlm.nih.gov/pubmed/11816953 [Accessed January 9, 2019].

Ramos, J. M., Toro, C., Reyes, F., Amor, A., and Gutiérrez, F. (2011). Seroprevalence of HIV-1, HBV, HTLV-1 and Treponema pallidum among pregnant women in a rural hospital in Southern Ethiopia. *J. Clin. Virol.* 51, 83–85. doi:10.1016/j.jcv.2011.01.010.

Saji, F., Tokugawa, Y., Kamiura, S., Samejima, Y., Ohashi, K., Azuma, C., et al. (1989). Vertical transmission of human T-cell leukemia virus type I (HTLV-I): detection of proviral DNA in HTLV-I carrier gravida. *J. Clin. Immunol.* 9, 409–14. Available at: http://www.ncbi.nlm.nih.gov/pubmed/2621244.

Sanchez-Palacios, C., Gotuzzo, E., Vandamme, A. M., and Maldonado, Y. (2003). Seroprevalence and risk factors for human T-cell lymphotropic virus (HTLV-I) infection among ethnically and geographically diverse Peruvian women. *Int. J. Infect. Dis.* 7, 132–137. doi:10.1016/S1201-9712(03)90009-9.

Sequeira, C. G., Tamegão-Lopes, B. P., dos Santos, E. J. M., Ventura, A. M. R., Moraes-Pinto, M. I., and Succi, R. C. de M. (2012). Estudo descritivo da infecção pelo HTLV em uma população de gestantes do Estado do Pará, norte do Brasil. *Rev. Soc. Bras. Med. Trop.* 45, 453–456. doi:10.1590/S0037-86822012005000007.

Souza, V. G. de, Martins, M. L., Carneiro-Proietti, A. B. de F., Januário, J. N., Ladeira, R. V. P., Silva, C. M. S., et al. (2012). High prevalence of HTLV-1 and 2 viruses in pregnant women in São Luis, state of Maranhão, Brazil. *Rev. Soc. Bras. Med. Trop.* 45, 159–162. doi:10.1590/S0037-86822012000200004.

Suzuki, S., Tanaka, M., Matsuda, H., Tsukahara, Y., Kuribayashi, Y., Gomibuchi, H., et al. (2014). Current status of HTLV-1 carrier in Japanese pregnant women. *J. Matern. Neonatal Med.* 27, 312–313. doi:10.3109/14767058.2013.814631.

Taylor, G. P., Bodéus, M., Courtois, F., Pauli, G., Del Mistro, A., Machuca, A., et al. (2005). The seroepidemiology of human T-lymphotropic viruses: Types I and II in Europe: A prospective study of pregnant women. *J. Acquir. Immune Defic. Syndr.* 38, 104–109. doi:10.1097/00126334-200501010-00018.

Tortevoye, P., Tuppin, P., Carles, G., Peneau, C., and Gessain, A. (2005). Comparative trends of seroprevalence and seroincidence rates of human T cell lymphotropic virus type I and human immunodeficiency virus 1 in pregnant women of various ethnic groups sharing the same environment in French Guiana. *Am. J. Trop. Med. Hyg.* 73, 560–5. Available at: http://www.ncbi.nlm.nih.gov/pubmed/16172481 [Accessed January 9, 2019].

Tortevoye, P., Tuppin, P., Peneau, C., Carles, G., and Gessain, A. (2000). Decrease of human T-cell lymphotropic virus type I prevalence and low incidence among pregnant women from a high endemic ethnic group in French Guiana. *Int. J. cancer* 87, 534–8. Available at: http://www.ncbi.nlm.nih.gov/pubmed/10918194 [Accessed January 28, 2019].

Trenchi, A., Gastaldello, R., Balangero, M., Irizar, M., Cudolá, A., and Gallego, S. (2007). Retrospective study of the prevalence of human T-cell lymphotropic virus-type 1/2, HIV, and HBV in pregnant women in Argentina. *J. Med. Virol.* 79, 1974–8. doi:10.1002/jmv.21027.

Treviño, A., Aguilera, A., Caballero, E., Toro, C., Eiros, J. M., Ortiz de Lejarazu, R., et al. (2009). Seroprevalence of HTLV-1/2 infection among native and immigrant pregnant women in Spain. *AIDS Res. Hum. Retroviruses* 25, 551–4. Available at: http://www.ncbi.nlm.nih.gov/pubmed/19544594 [Accessed January 28, 2019].

Treviño, A., Benito, R., Caballero, E., Ramos, J. M., Parra, P., Roc, L., et al. (2011). HTLV infection among foreign pregnant women living in Spain. *J. Clin. Virol.* 52, 119–122. doi:10.1016/j.jcv.2011.06.010.

Tsuji, Y., Doi, H., and Yamabe, T. (1990). Prevention of Mother-to-Child Transmission Human T-Lymphotropic Virus Type-I. *Pediatrics* 86, 11–17.

Tuppin, P., Lepère, J. F., Carles, G., Ureta-Vidal, A., Gérard, Y., Peneau, C., et al. (1995). Risk factors for maternal HTLV-I infection in French Guiana: high HTLV-I prevalence in the Noir Marron population. *J. Acquir. Immune Defic. Syndr. Hum. Retrovirol.* 8, 420–5. Available at: http://www.ncbi.nlm.nih.gov/pubmed/7882109 [Accessed January 28, 2019].

Tuppin, P., Makuwa, M., Guerma, T., Bazabana, M. M., Loukaka, J. C., Jeannel, D., et al. (1996). Low HTLV-I/II seroprevalence in pregnant women in Congo and a geographic cluster of an HTLV-like indeterminate western blot pattern. *J. Acquir. Immune Defic. Syndr. Hum. Retrovirol.* 11, 105–7. Available at: http://www.ncbi.nlm.nih.gov/pubmed/8528728 [Accessed January 28, 2019].

Udeze, A. O., Odebisi-Omokanye, M. B., Faneye, A., Olusola, B. A., Ogunsemowo, O., Iwuoha, C., et al. (2018). Serological detection of human T-cell lymphotropic virus types I and II among pregnant women in Ilorin, Nigeria. *J. Immunoass. Immunochem.* 39, 428–438. doi:10.1080/15321819.2018.1500374.

Verdier, M., Denis, F., Sangaré, A., Barin, F., Gershy-Damet, G., Rey, J. L., et al. (1989). Prevalence of antibody to human T cell leukemia virus type 1 (HTLV-1) in populations of Ivory Coast, West Africa. *J. Infect. Dis.* 160, 363–70. Available at: http://www.ncbi.nlm.nih.gov/pubmed/2547879 [Accessed January 28, 2019].

Wiktor SZ, Pate EJ, Murphy EL, Palker TJ, Champegnie E, Ramlal A, Cranston B, Hanchard B, B. W., Wiktor, S. Z., Pate, E. J., Murphy, E. L., Palker, T. J., Champegnie, E., et al. (1993). Mother-to-child transmission of human T-cell lymphotropic virus type I (HTLV-I) in Jamaica association with antibodies to envelope glycoprotein (gp46) epitopes. *J. Acquir. Immune Defic. Syndr.* 6, 1162–1167. Available at: http://www.ncbi.nlm.nih.gov/pubmed/7692038.

Yamada, T., Togashi, T., Tsutsumi, H., Imamura, M., Okubo, H., Okabe, M., et al. (2014). Prevalence of human T-lymphotropic virus type 1 carriers among pregnant women in Hokkaido, Japan. *Microbiol. Immunol.* 58, 427–431. doi:10.1111/1348-0421.12165.

Ydy, R. R. A., Ferreira, D., Souto, F. J. D., and Fontes, C. J. F. (2009). Prevalência da infecção pelo vírus linfotrópico humano de células T - HTLV-1/2 entre puérperas de Cuiabá, Estado de Mato Grosso, 2006. *Rev. Soc. Bras. Med. Trop.* 42, 28–32. doi:10.1590/S0037-86822009000100007.

Zurita, S., Costa, C., Watts, D., Indacochea, S., Campos, P., Sanchez, J., et al. (1997). Prevalence of human retroviral infection in Quillabamba and Cuzco, Peru: a new endemic area for human T

cell lymphotropic virus type 1. *Am. J. Trop. Med. Hyg.* 56, 561–5. Available at: http://www.ncbi.nlm.nih.gov/pubmed/9180608 [Accessed January 28, 2019].
